# Exploring Cellulose Triacetate Nanofibers as Sustainable Structuring Agent for Castor Oil: Formulation Design and Rheological Insights

**DOI:** 10.3390/gels10040221

**Published:** 2024-03-25

**Authors:** M. A. Martín-Alfonso, José F. Rubio-Valle, Gethzemani M. Estrada-Villegas, Margarita Sánchez-Domínguez, José E. Martín-Alfonso

**Affiliations:** 1 Chemical Product and Process Technology Research Center (Pro2TecS), Department of Chemical Engineering and Materials Science, University of Huelva, 21071 Huelva, Spain; manuelantonio.martin@diq.uhu.es (M.A.M.-A.); josefernando.rubio@diq.uhu.es (J.F.R.-V.); 2 CONACYT-Centro de Investigación en Química Aplicada, Parque de Innovación e Investigación Tecnológica (PIIT), Apodaca 66628, Mexico; mayeli.estrada@ciqa.edu.mx; 3 Centro de Investigación en Materiales Avanzados, S.C. (CIMAV), Unidad Monterrey, Alianza Norte 202, Apodaca 66628, Mexico; margarita.sanchez@cimav.edu.mx

**Keywords:** green products, triacetate cellulose, castor oil, electrospinning, oil structuring, rheology

## Abstract

Developing gelled environmentally friendly dispersions in oil media is a hot topic for many applications. This study aimed to investigate the production of electrospun cellulose triacetate (CTA) nanofibers and to explore their potential application as a thickening agent for castor oil. The key factors in the electrospinning process, including the intrinsic properties of CTA solutions in methylene chloride (DCM)/ethanol (EtOH), such us the shear viscosity, surface tension, and electrical conductivity, were systematically studied. The impact of the CTA fiber concentration and the ratio of DCM/EtOH on the rheological properties of the gel-like dispersions in castor oil was then investigated. It was found that dispersions with a non-Newtonian response and above a critical concentration (5 wt.%), corresponding to approximately 2–2.5 times the entanglement concentration, are required to produce defect-free nanofibers. The average fiber diameter increased with CTA concentration. Further, the morphology and texture of the electrospun nanofibers are influenced by the ratio of solvents used. The rheological properties of dispersions are strongly influenced by the concentration and surface properties of nanofibers, such as their smooth or porous textures, which allow their modulation. Compared to other commonly used thickeners, such as synthetic polymers and metal soaps, CTA electrospun nanofibers have a much higher oil structuring capacity. This work illustrated the potential of using CTA nanofibers as the foundation for fabricating gel-like dispersions in oil media, and thus exerting hierarchical control of rheological properties through the use of a nanoscale fabrication technique.

## 1. Introduction

Nanostructured materials in the form of fiber structures exhibit properties different from their bulk counterparts at the micro- and nanometer scale, and are highly versatile in application [1,2]. Recently, there has been an increased demand for biodegradable and eco-friendly fibers due to growing concerns about sustainability and environmental protection [3]. Among these renewable resources, cellulose has gained more attention due to its abundance and notable characteristics. Cellulose derivatives, such as ethyl cellulose, cellulose acetate, and carboxymethyl cellulose, can be used for the design of functional nanofibers. This offers the possibility of making nanostructured materials with promising functionalities, renewability, flexibility, and biodegradability [4,5]. Among them, cellulose triacetate (CTA) is a polymer produced by chemically reacting natural cellulose with acetic anhydride. The hydroxyl groups in cellulose have a substitution degree of 2.7–3.0. CTA is known for its high transparency, excellent solvent resistance, and heat resistance, making it a highly desirable polymer. CTA can be easily processed from melt or solution into films, membranes, and fibers, making it an important cellulose ester in membrane technology [6,7]. In addition, triacetate cellulose nanocomposites have recently been obtained through thermomechanical processes or pervaporation methods, demonstrating unique properties for various engineering applications [8,9]. However, the potential of electrospun micro-/nanofiber mats made of triacetate cellulose, which exhibit a high specific surface area and porosity, has not been thoroughly studied.

Electrospinning is a versatile, simple, and efficient technique for producing polymer micro-/nanofibers. The technique allows for the control of fiber morphology by adjusting various parameters, which can be classified into two main categories: solution parameters (such as the concentration, molecular weight, surface tension, conductivity, and viscosity) and operating parameters (such as the voltage, distance between the tip and collector, flow rate, ambient humidity, and temperature) [10]. Several studies have shown that electrospinnability is closely related to both the electrospinning process parameters and the physicochemical properties of the spinning solutions, particularly the surface tension, electrical conductivity, and viscosity [11,12]. These physicochemical properties depend on the type of polymer, the solvents used, and the concentration of polymer used to formulate the solution [13]. Hence, electrospinning technology can produce a variety of nanostructures with multifunctional properties, including particles, beaded fibers, smooth fibers, and ribbons, making them suitable for numerous applications. Previous works have explored the electrospinnability of cellulose triacetate [14,15]. Han et al. [14] studied the effect of a mixed solvent of methylene chloride (DCM)/ethanol (EtOH) on the surface morphology and diameter distribution of cellulose triacetate fibers, and found that CTA electrospun fibers with MC and MC/EtOH (90/10 *v*/*v*) had pores with a narrow size distribution, while non-porous corrugated fibers were obtained from MC/EtOH (80/20 *v*/*v*) due to their lower vapor pressure. Lan et al. [15] investigated the changes in size and morphology of electrospun fiber mats made of cellulose triacetate in a binary dimethylsulfoxide (DMSO)/chloroform system. It was found that CTA fibers with diameters ranging from 98 nm to 1.81 μm were obtained from 8 wt.% CTA solutions in all DMSO/chloroform solvent systems. Additionally, it was observed that the average diameter of CTA nanofibers decreased, and the size distribution narrowed as the DMSO content in the mixed solvent increased. These results indicated that the electrospinning process and the morphology of cellulose triacetate nanofibers were significantly affected by the solvent type and the polymer concentration of biopolymer.

On the other hand, the use of different types of thickeners for oil structuring has gained significant attention in industry and academia over the last few years. This is particularly true for food applications, where it is considered to be a promising strategy for the replacement of fat [16]. It has also been explored in the pharmaceutical [17] and lubricant [18,19] industries. Particularly in the lubricant industry, the semi-solid lubricants market is primarily dominated by products based on metal soaps or petrochemical-derived polymers. These products are not environmentally friendly and require complex production processes. Hence, creating technologically efficient oil thickeners from natural polymers presents a significant challenge in terms of environmentally friendly alternatives. Gel-like dispersions are formed using a single thickener or a combination of different thickener molecules that create an entanglement network, trapping the oil into its micro- and nanostructure. The mechanical and rheological properties of gel-like dispersions depend on the relationship among its components, such as gelators, oils, and surfactants, and the structuring mechanisms produced during manufacturing [20]. Several biopolymers can gel oils by forming supramolecular structures through physical entanglements or chemical crosslinking among polymer chains [21]. These interactions must balance solvent–thickener and thickener–thickener interactions.

**Figure 1:**
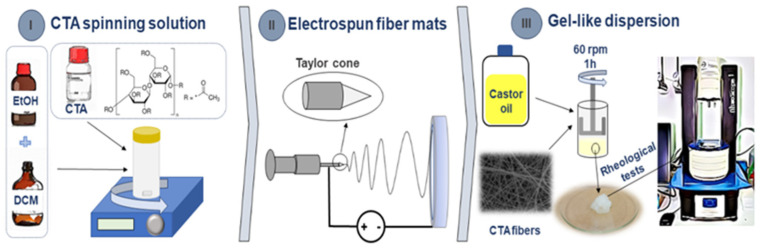
Scheme of the manufacturing process for CTA gel-like dispersions.

The physical structuring of thickened oils is normally achieved by raising the temperature of the system mixture above the glass transition temperature of the thickener and then cooling it to room temperature, a process known as thermo-gelation. However, this procedure does not allow for the complete or partial monitoring of the microstructure of the thickener, including its physicochemical and mechanical properties. Therefore, innovative approaches for incorporating thickeners into an oil phase that enables true control of the three-dimensional network of gel-like dispersions would be highly beneficial for many applications. In this sense, recent works have described a new method of structuring vegetable oils using electrospun nanostructures as an alternative approach [22,23,24]. This method offers a novel way to structure vegetable oils, which could have significant implications in fields such as pharmaceuticals, cosmetics, food technology, and lubricants. A combination of design rules underpins the hypothesis of this study, which also provides the novelty and value added of this research: (i) the electrospun nanostructures’ high porosity, nanometric size, and high surface/volume ratio may enable the formation of a distinctive three-dimensional network that enhances the fibers’ physical interactions with the oil; (ii) from a chemical point of view, the CTA biopolymer with a high degree of substitution of the acetyl group could provide an adequate compatibility with castor oil to be used as a potential thickener. Taking into account these considerations, this work focused on the development of gel-like dispersions, using electrospun CTA fibers as the thickener agent. To achieve this goal, the influence of the physicochemical and shear rheological properties of CTA solutions on its electrospinnability was studied. Then, the rheological properties of the resulting gel-like dispersions were comprehensively evaluated, taking into account the impact of the fiber concentration and surface properties. One scheme of the manufacturing process from the electrospinning of CTA solutions to the production and rheological characterization of CTA gel-like dispersions is shown in Figure 1.

## 2. Results and Discussion

### 2.1. Physico-Chemical Properties of Cellulose Triacetate Solutions

**Figure 2:**
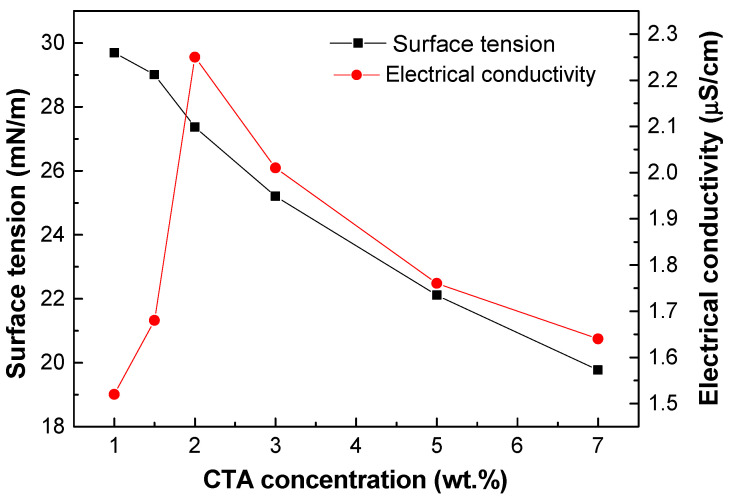
Surface tension and electrical conductivity for CTA spinning solutions in 7/3 DM/EtOH as function of concentration.

Figure 2 shows the surface tension and electrical conductivity of the solutions as a function of the CTA concentrations in a mixed solvent of 7/3 DCM/EtOH. The surface tension values decreased with increasing CTA concentration, ranging from 29.01 to 19.77 mN/m. This decrease is consistent with other studies and supports the formation of uniform electrospun nanofibers [22,23,25]. This is due to several factors. Firstly, by reducing the surface tension, smaller droplets are formed, facilitating the production of more uniform fibers [13,26]. In addition, this reduction allows for greater elongation before the droplet breaks, improving the stability of the polymer solution jet during the process [10,27]. The electrical conductivity started to increase with CTA concentration for the systems prepared at 1.5 and 2 wt.%, and for the rest of the prepared solutions it gradually decreased with increasing concentration. This decrease is in agreement with previous studies performed by other authors, such as Sanchez-Cid et al. [28], where they investigated the influence of the solution properties on the electrospinning process of different cellulose derivatives. The decrease can be attributed to a lower mobility of the entangled macromolecules at high concentrations of biopolymers because they present concentrations above the overlapping concentration [22,29].

**Figure 3:**
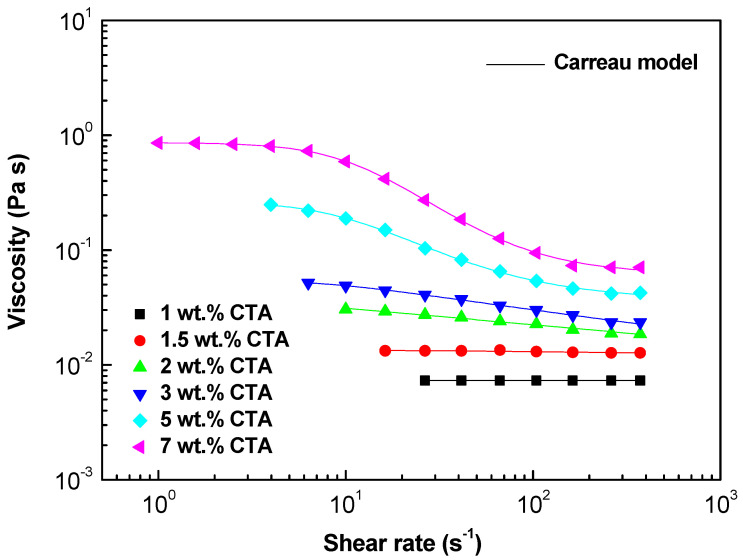
Viscous flow curves vs. concentration plot for CTA spinning solutions in 7/3 DM/EtOH as function of concentration.

Figure 3 shows the flow curves (apparent viscosity versus shear rate) of CTA solutions in 7/3 DCM/EtOH as a function of CTA concentration. The solutions prepared at 1 and 1.5 wt.% showed Newtonian behavior throughout the shear rate range studied. Nevertheless, as the CTA concentrations increased, the viscous flow behavior transitioned to shear thinning above a critical shear rate. This flow behavior can be observed to be characterized by three distinct regions: (i) a Newtonian region at low shear rates ($\eta_{0}$); (ii) a subsequent shear thinning region at intermediate shear rates; and (iii) a tendency to stabilize at a constant high shear rate, limiting the viscosity ($\eta_{\infty }$) [23]. The viscosity’s dependence on the shear rate aligns quite well with the Carreau model [30]. 

The parameters of the fits to the Carreau model are shown in Table 1. It can be seen that the values of $\eta_{0}$, $\eta_{\infty }$, and p show a direct correlation with increasing CTA concentration in the electrospinnable solutions as they gradually increased. These results are in agreement with those reported by the authors of [31,32], and especially with the work of Han et al. [33], who emphasized the importance of studying the rheological properties of solutions to obtain defect-free fibers. To this end, they studied the tunable effects of ε-polysin in improving the electrospinning equipment and obtaining defect-free fibers for ultra-high molecular weight polyacrylamide by characterizing the properties of solutions and mats’ electrospun morphologies.

**Figure 4:**
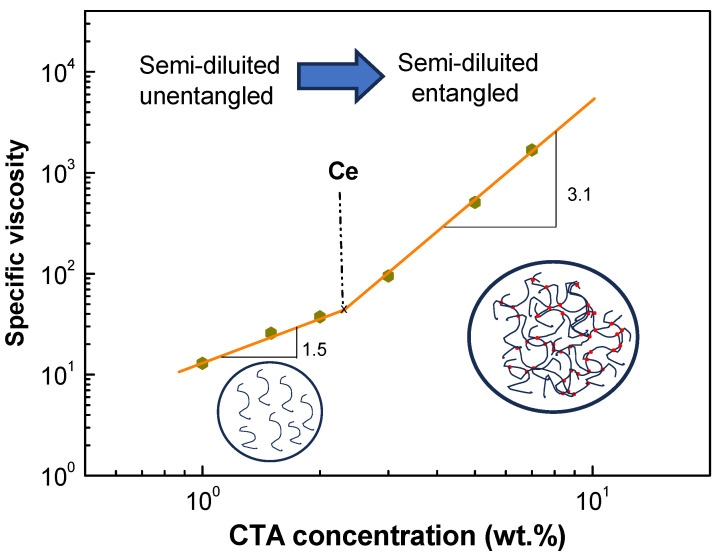
Plot of specific viscosity (*η_sp_*) as a function of CTA concentration.

The solution viscosity, which measures polymer entanglement, can predict the fiber formation during electrospinning. Different polymer concentration regions have been correlated with common fiber morphologies, such as beaded fibers and uniform fibers. Polymer viscosity–concentration relationships have been measured by various authors [34,35]. Different concentration regimes have been identified, including dilute, semi-dilute unentangled, semi-dilute entangled, and concentrated regimes. Several boundaries between concentration regimes have been identified: (i) the chain overlap concentration, C*, is the point where the dilute and semi-dilute unentangled regimes intersect; (ii) the entanglement concentration, Ce, is the point where the semi-dilute unentangled and semi-dilute entangled regimes intersect [23]. At this point, the macromolecular chain motion is constrained by a significant overlap of the polymer chains topologically. Figure 4 displays the correlation between the specific viscosity (*η_sp_*, Equation (1)) and the CTA concentration.

(1) $$\eta_{sp}=\frac{\eta_{ps}-\eta_{sol}\;}{\eta_{sol}}=\eta_{rel}-1$$

where *η_sol_* and *η_ps_* refer to the viscosities of the solvent, 70/30 DCM/EtOH in this instance, and the CTA spinning solutions. Figure 4 displays the correlation between the specific viscosity and the CTA concentration [28,36]. The critical entanglement concentration (Ce), which separates the semi-diluted unentangled and semi-diluted entangled regimes, can be determined by observing the change in the slope on this graph [12,37]. As observed, Ce was approximately 2.3 wt%, beyond which there was an escalation in the scaling exponent, transitioning from *η_sp_* α C^1.5^ to *η_sp_* α C^3.1^, which is consistent with expected values for a neutral polymer in a favorable solvent [38]. Ce can be used to roughly estimate the suitability of polymer solutions for electrospinning and to predict the morphology of the resulting electrospun structures [12,28]. It has been recommended that the solution concentration should be at least 2–2.5 times Ce [39,40,41]. For instance, CTA spinning solutions with concentrations around 5 wt.% are more suitable for producing uniform free bead-fibers. In addition, these experimental results support previous hypotheses regarding the effect of the concentration on conductivity, demonstrating a decrease above 2 wt.% due to reduced macromolecular mobility within the semi-dilute entanglement regime [12,22].

To assess the characteristics of the dilution states within these solutions and offer deeper insights into polymer–solvent interactions, we conducted a hydrodynamic investigation. The intrinsic viscosity, a parameter indicative of the capacity of macromolecules to enhance the solution viscosity without intermolecular interactions [42], was calculated and scrutinized in relation to the concentration using established relationships [43,44].

(2) $$\eta_{red}=\frac{\eta_{sp}}{C}\;\;\;\;$$

(3) $$\left[\eta \right]=\underset{C\to 0}{\lim}\;\frac{\eta_{sp}}{c}$$

where the terms *η_rel_* and *η_red_* denote the relative and reduced viscosities, respectively (Equations (1) and (2)). Finally, [*η*] stands for the intrinsic viscosity. The most commonly used methods for estimating intrinsic viscosity are based on the Kraemer and Huggins models, as expressed using Equations (4) and (5), respectively.

(4) $$\frac{\ln\eta_{rel}}{c}=\left[\eta \right]+k_{1}{\left[\eta \right]}^{2\;}c$$

(5) $$\eta_{red}=\;\left[\eta \right]+k_{2}\;{\left[\eta \right]}^{2}\;c$$

**Figure 5:**
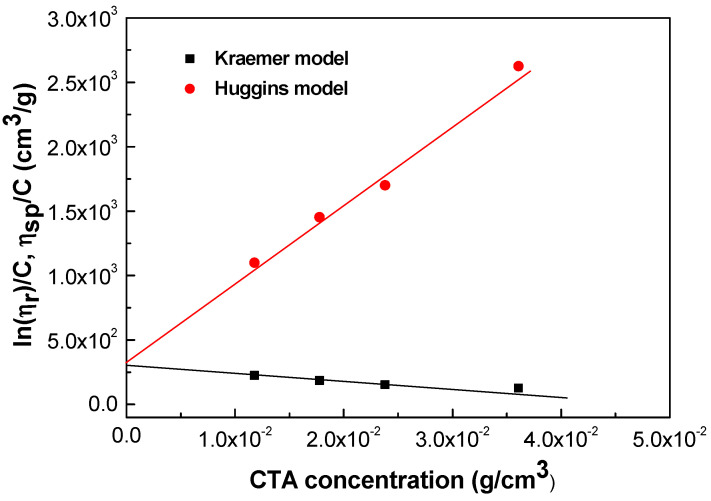
Kraemer and Huggins plots for CTA solution concentration.

To determine the relative and reduced viscosities of CTA solutions, Equations (1) and (2) were used and the results were plotted according to Equations (4) and (5), as shown in Figure 5 for concentrations ranging from 1 to 7 wt%. The intrinsic viscosity was then extrapolated as the Y-intercept corresponding to a zero concentration [45]. Both models showed good fits to the experimental data, yielding intrinsic viscosity values between 301 and 307 cm^3^/g. Intrinsic viscosity is known to provide an insight into the interactions of individual polymer molecules with the solvent and the polymer-specific hydrodynamic volume or average molecular weight [46]. The high intrinsic viscosity values indicated compact CTA structures in DCM/EtOH with good interactions with the solvent [38]. This indicates an excellent compatibility between CTA and DCM/EtOH. 

On the other hand, intrinsic viscosity values can be used to determine the molecular weight using the Mark–Houwink–Sakurada equation (Equation (6)) [47]:

(6) $$\left[\eta \right]=\;K\;\times M^{\alpha }$$

where *K* is the proportionality constant in the Mark–Houwink–Sakurada equation. Its value depends on the specific polymer–solvent system and reflects the interaction between polymer and solvent values. On the other hand, *α* is known as the power law exponent; both *K* and *α* are determined experimentally for each polymer–solvent system. Kamide et al. [48] estimated the viscosities of the molecular parameters, performed rheological and light scattering measurements on twelve fractions of CTA (acetic acid content, 61.0 wt.%) using various solvents including DCM/EtOH mixtures, and determined the values of *K* and *α* empirically. Using the values of *K* and *α* (1.41 × 10^−2^ and 0.834, respectively) and the previously calculated intrinsic viscosity, and substituting in Equation (6), a viscosity average molecular mass of 2.56 × 10^4^ g/mol was obtained, which is within the range empirically determined by the authors of [48]. Finally, the intrinsic viscosity can be related to the Ce, which helps to confirm that the rheological measurements and calculations have been performed correctly. Graessley proposed an equation derived from de Gennes’s repatterning theory relating intrinsic viscosity to Ce [49]. Using this derivation, a Ce ≈ 2.13 wt.% was obtained, which is very similar to that obtained empirically in Figure 4.

### 2.2. Characterization of Cellulose Triacetate Electrospun Nanostructures

**Figure 6:**
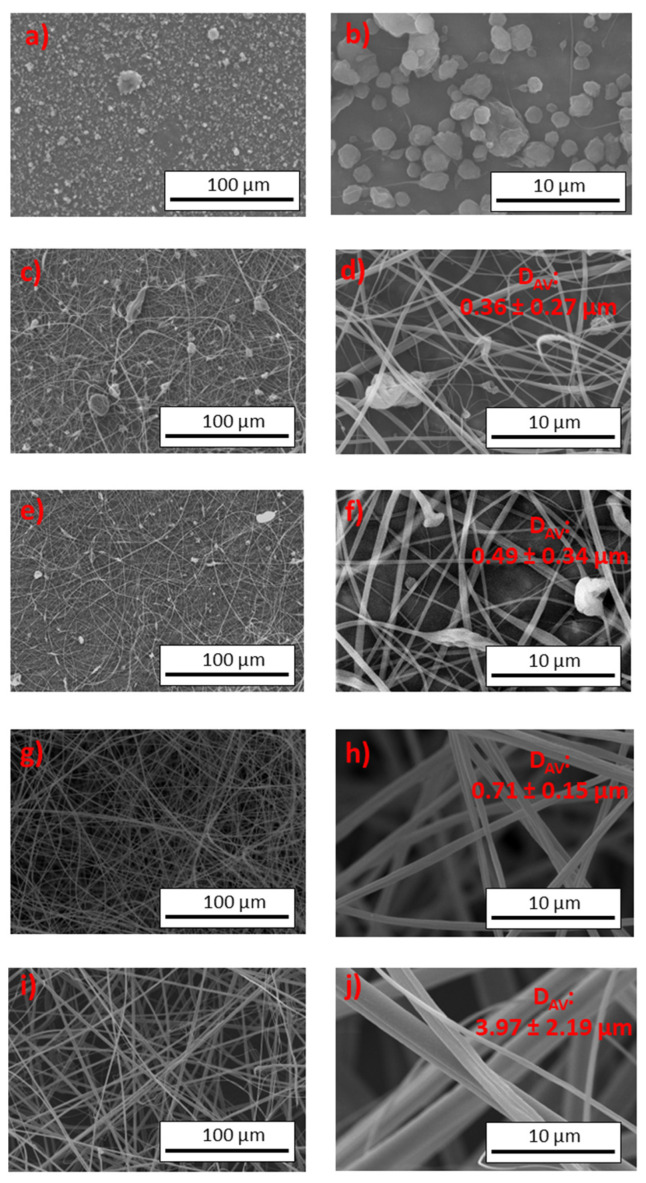
SEM micrographs obtained for different concentrations of CTA spinning solutions in 7/3 DCM/EtOH: (**a**,**b**) 1 wt.%, (**c**,**d**) 2 wt.%, (**e**,**f**) 3 wt.%, (**g**,**h**) 5 wt.%, and (**i**,**j**) 7 wt.%.

Figure 6 shows SEM micrographs of different electrospun nanostructures obtained from CTA solutions at different concentrations using 7/3 DCM/EtOH. The solution prepared at 1 wt.% failed to generate fibers or interconnected fibers, which is a physical electrospray phenomenon. Consequently, the image consists of agglomerated microparticles (see Figure 6a,b). Slightly increasing the solution concentration to 2 wt.% (Figure 6c,d) resulted in morphologies with micro- and nanosized particles interconnected by fine filaments (approximately 360 nm in diameter). A similar morphology (fiber with some large particles) was observed for 3 wt.%, but with a higher number of fibers (Figure 6e,f), with an average diameter of 490 ± 340 nm. However, from the 5 wt.% solution, consistent mats were obtained with uniform nanofibers whose diameter increased with concentration, with an average diameter of 710 ± 150 nm (Figure 6g,h). For the system prepared at 7 wt.% (Figure 6i,j), smooth fibers with some parallel lines on the fiber surface were obtained, with an average fiber diameter of 3971 ± 32,190 nm. Huang et al. [50] also found a similar result for cellulose acetate butyrate fibers. These results were consistent with the hypotheses previously mentioned in Figure 4 and Figure 5. Solutions with concentrations in the semi-dilute unentangled range produced particles or particle aggregates, whereas solutions in the semi-dilute entangled range produced fibers with high uniformity. CTA solutions with concentrations above 5 wt.% (~2–2.5 times Ce) produced uniform fibers, while solutions with concentrations around 2 wt.%, approaching the estimated Ce, produced predominantly interconnected particles with nanofibers. Furthermore, these results were consistent with those reported by other authors who have studied the electrospinnability of polymers as a function of spinning solution concentration and their resulting physicochemical properties [22,51,52].

**Figure 7:**
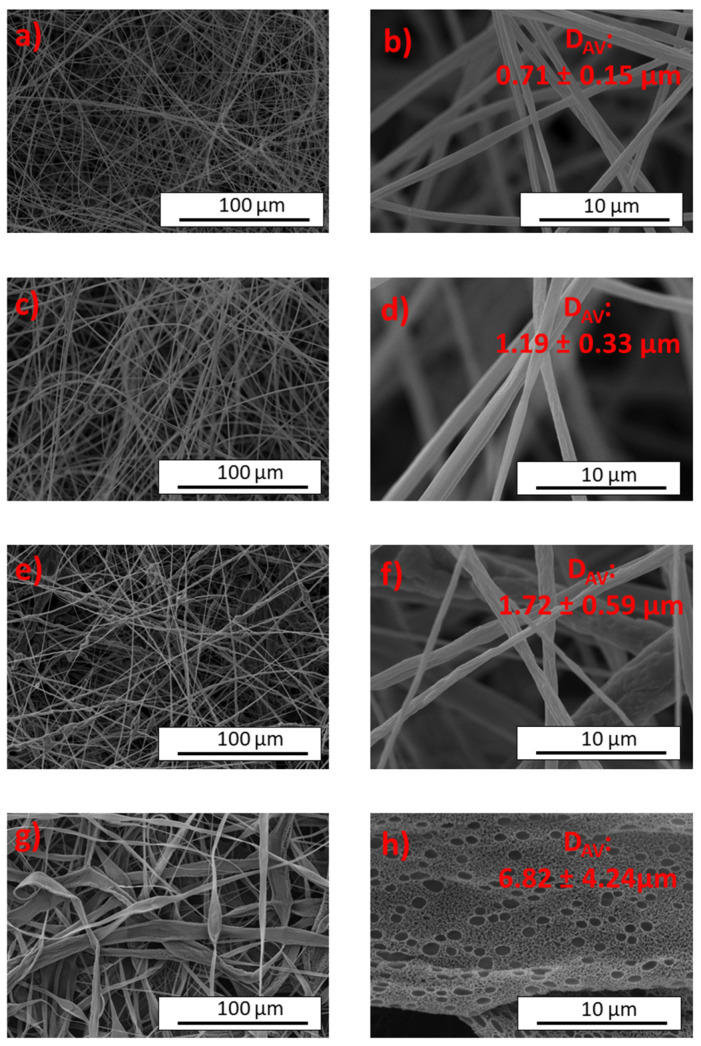
SEM micrographs of structures obtained from polymer solutions containing 5 wt.% CTA concentration at different ratios of DCM/EtOH: (**a**,**b**) 7/3, (**c**,**d**) 8/2, (**e**,**f**) 9/1, and (**g**,**h**) 10/0.

To investigate the effect of DCM/EtOH solvents, we used electrospun nanofibers with a 5 wt.% concentration of CTA as a reference system and examined the effect of varying DCM/EtOH ratios on the nanofibers (Figure 7). Starting from the initial 7/3 DCM/EtOH in the reference system, we produced solid fibers with an average diameter of 0.71 µm and some parallel lines on the fiber surface. Increasing the DCM content in the binary solvent system resulted in larger fiber diameters for the 8/2 DCM/EtOH (Figure 7c,d, average diameter of 1.19 µm). However, we also observed an irregular surface on these electrospun fibers. This irregularity became more pronounced when a 9/1 DCM/EtOH was used (see Figure 7e,f), resulting in irregular and uneven fibers with some bead formation. In Figure 7g,h, the micrographs show an electrospun mat obtained using pure DCM as solvent, which shows relatively flat porous ribbons with some inhomogeneity and two different pore sizes (average size of 0.89 µm for the larger ones). This porous nature can be attributed to the higher evaporation rate of DCM compared to EtOH [53]. Similar phenomena have been reported and discussed in previous studies [54,55], suggesting that as the solvent absorbs energy to overcome the vapor pressure in order to evaporate, the surface temperature of the fibers in the formation is lowered, a phenomena known as evaporative cooling; this generates the condensation of water droplets onto the forming fibers that contribute to pore formation when water is eliminated [54]. In addition, the needle tip can plasticize during the electrospinning process, leading to clogging in the hydraulic system, which poses significant challenges and affects the morphology of the electrospun mats [22,56]. In addition, the evaporation of DCM and its lower viscosity also contribute to the irregular texture of these structures, with small pores that may not be visible in our photographs. In summary, changing the solvent ratio, especially increasing the DCM content, results in a transition from relatively defect-free fibers to irregular and uneven flat ribbons [57], accompanied by an increase in the fiber and pore size [53].

**Figure 8:**
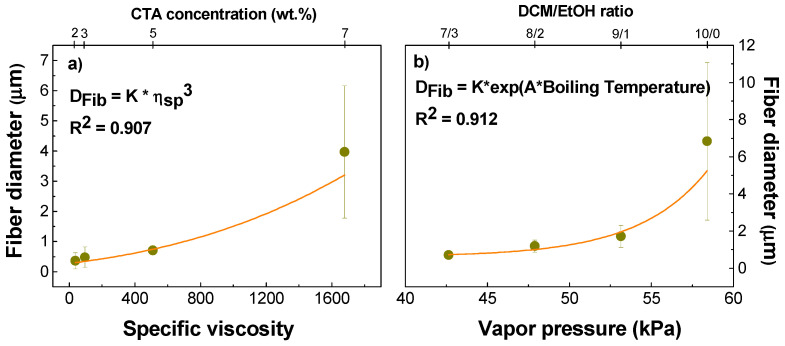
Relationships between the average fiber diameter and (**a**) the specific viscosity (*η_sp_*) of CTA solutions in 7/3 DCM/EtOH, and (**b**) the vapor pressure of the different DCM/EtOH ratios.

Figure 8a shows the correlation between the average fiber size obtained and the specific viscosity. It can be seen that there is a clear relationship between the specific viscosity of the solution and the average fiber diameter, represented by an empirical power law. Conversely, Figure 8b illustrates the relationship between the average fiber size and the different boiling temperatures obtained by using different DCM/EtOH ratios at the standard concentration of 5 wt.%. In particular, an empirical exponential relationship between the boiling temperature and fiber diameter is observed, thus confirming the above hypothesis.

### 2.3. Ability of Cellulose Triacetate Nanostructures to form Gel-like Dispersions

**Figure 9:**
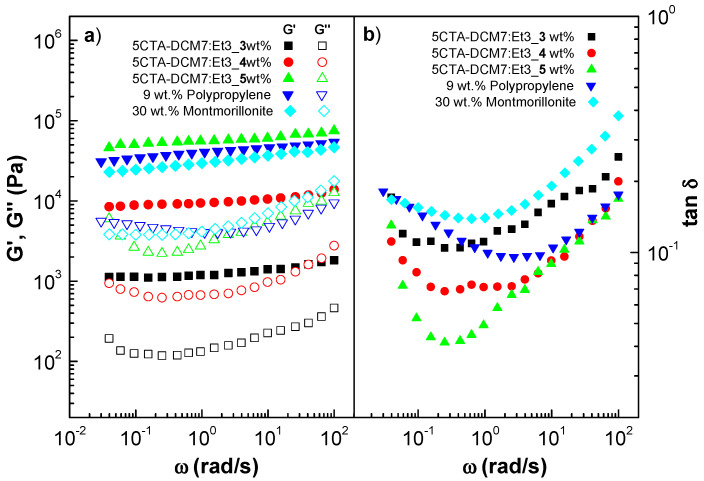
Frequency dependence of the storage and loss moduli (**a**) and loss tangent (**b**) in the linear viscoelastic region for gel-like dispersions as a function of thickener content. Polypropylene and montmorillonite oleogels are included for the sake of comparison.

Figure 9 shows the mechanical spectra of gel-like dispersions as a function of thickener concentration while keeping the concentration of spinning solution at 5 wt.% constant. For comparison purposes, the mechanical spectra of the well-known oleogels formulated with polypropylene and montmorillonite are also displayed [19,58]. As can be seen, the linear viscoelastic response was qualitatively similar for all the samples studied. This behavior corresponded to the so-called plateau relaxation zone. This region is characterized by the fact that the storage modulus (G′) is higher than the loss modulus (G″) over the entire frequency range covered, with both moduli following a different pattern depending on frequency. G′ increases slightly with frequency, while G″ shows a clear minimum. This region is characteristic of the occurrence of physical entanglements in the microstructural network, in this case due to the interaction between CTA fibers and castor oil. Moreover, these mechanical spectra are qualitatively similar to other polypropylene and montmorillonite oleogels previously studied, and are also very similar to those shown by conventional lithium lubricating greases, with G′ values typically in the range of 10^4^ to 10^5^ Pa at 25–75 °C, approximately an order of magnitude higher than G″ values, depending on the composition and processing conditions [59]. As can be observed in Figure 9a, the values of G′ and G″ increased with the thickener concentration, indicating that the fiber density in the percolation network increased, which was associated with packing effects. In addition, the loss tangent, defined as the relationship between G″ and G′, was also dependent on the CTA thickener content over the entire frequency range studied (Figure 9b), indicating that the relative elastic properties of dispersion increase as the CTA thickener content decreases. 

**Figure 10:**
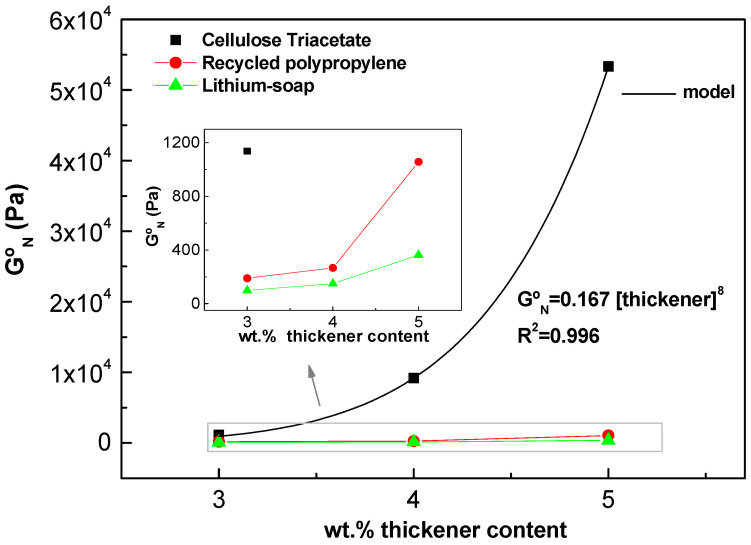
Evolution of plateau modulus of gel-like dispersions as a function of thickener content.

Figure 10 presents a summary of the impact of the thickener content on the linear viscoelastic response of gel-like dispersions, as demonstrated by the evolution of the ‘plateau’ modulus (G_N_^0^). Values for other gel-like dispersions formulated with different thickeners, such as recycled polypropylene (rec-PP) and lithium soap (Li-soap), are also shown for comparison, and were adapted from our previous works [60,61]. The plateau modulus is a parameter that characterizes the ‘plateau’ relaxation zone in the mechanical spectrum. It is described elsewhere [62] and can be considered a measure of the density of interparticle interaction in the microstructural network. Therefore, it is related to the strength the microstructural network, and in this case, it may be attributed to the packing effect of the percolation network formed among electrospun fibers. A simple method of estimating G_N_^0^ from the loss tangent was selected in this work [63]:

(7) $${G_{N}}^{o}={\left[G\prime \right]}_{\tan\;\delta \to minimum}$$

It is evident that the plateau modulus increases with CTA fibers concentration, and it can be accurately modelled using a power law equation:

(8) $${G_{N}}^{o}=a\cdot {\left[wt.\%\;fibers\right]}^{b}$$

where *a* and *b* are fitting parameters. Interestingly, CTA electrospun fiber has a much higher thickening capacity for structuring oil compared to other commonly used thickeners in lubricant applications, such as synthetic polymers and metal soaps. This finding could be important from both technical and economic perspectives, not only for this application but also for others.

**Figure 11:**
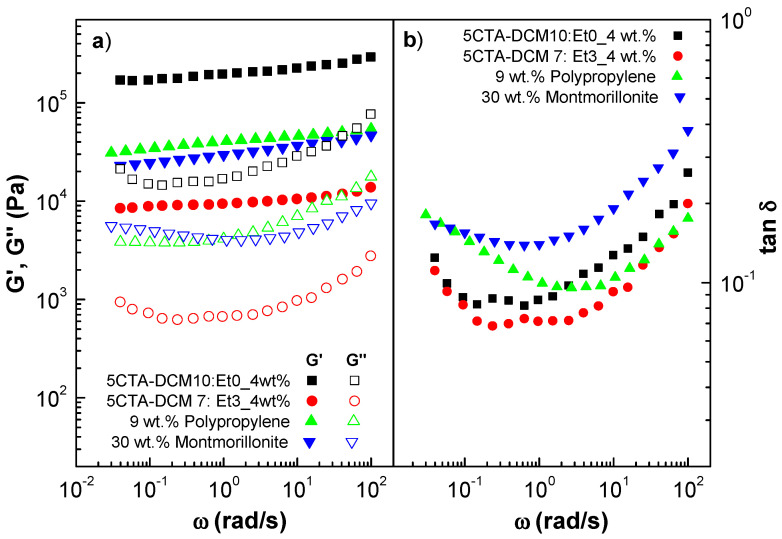
Frequency dependence of the storage and loss moduli (**a**) and loss tangent (**b**) in the linear viscoelastic region for gel-like dispersions as a function of solvent ratio. Polypropylene and montmorillonite oleogels are included for the sake of comparison.

Figure 11 shows the mechanical spectra of gel-like dispersions formulated with fibers obtained from polymer solutions containing a 5 wt.% CTA concentration and different binary solvent ratios (7/3 DCM/EtOH and 10/0 DCM/EtOH). As can be observed, the linear viscoelastic response was qualitatively similar to all the gel-like dispersions studied above and to the oleogels formulated with polypropylene and montmorillonite (G′ > G″ and a minimum in tan δ). It should be noted that the surface properties of the fibers have a great influence on the viscoelastic properties of the gel-like dispersions. Gel-like dispersions prepared with porous fibers gave viscoelastic modulus values approximately a decade higher than those of dispersions formulated with smooth fibers, with similar relative elastic behavior.

## 3. Conclusions

The development of gel-like dispersions in oil media with environmentally friendly and functional properties is a cutting-edge research area for many applications in a wide range of industries such as pharmaceuticals, cosmetics, food, and lubricants. Formulation design and synthesis protocol are crucial aspects to consider. In this work, cellulose triacetate (CTA) nanofibers were produced using electrospinning in DCM/EtOH solutions and validated the hypothesis initially proposed as castor oil structuring agents in this research. The experimental shear rheology and surface tension data suggest that the spinnability of CTA solutions is correlated with the production of uniform fibers. Uniform nanofibers without beads were produced from CTA solutions that showed a non-Newtonian response with a minimum zero-shear rate, limiting the viscosity to ~0.265 Pa∙s. For CTA concentrations above 5 wt.%, these requirements were met. This concentration corresponded to 2–2.5 times the entanglement concentration, Ce. However, particles and nanofibers with beads morphologies were obtained from solutions with concentrations lower than Ce and slightly higher than Ce in the semi-diluted and entangled regime, respectively. The electrospinnability of CTA solutions was improved by an increase in the polymeric chain entanglements and a decrease in the surface tension. Additionally, the morphology and texture of the electrospun nanofibers were greatly influenced by the solvent ratio, particularly its volatility. 

Gel-like dispersions that are physically stable were produced using electrospun CAb nanofibers and castor oil. The rheological properties of dispersions are strongly influenced by the concentration and surface properties of nanofibers, such as their smooth or porous textures, which allow for their modulation. A higher concentration of nanofibers and porous fibers resulted in increased thickening properties due to the greater interactions between the fibers and the oil. Compared to other commonly used thickeners, such as synthetic polymers and metal soaps, CTA electrospun nanofibers have a much higher oil structuring capacity. 

In summary, the electrospun CTA nanofibers have the capability of oil structuring, which creates new opportunities in various fields. This allows for the development of innovative and bio-based functional thickening agents or additives, contributing to the creation of fully renewable products.

## 4. Materials and Methods

### 4.1. Materials

Cellulose triacetate (M_n_ 5 × 10^4^ g/mol and DS 2.92) [64,65] was used along with dichloromethane (DCM, purity 99.8%) and ethanol (EtOH, purity 99.9%) purchased from Merck Sigma-Aldrich (Taufkirchen, Germany) as raw materials for preparing polymer solutions for electrospinning. Castor oil from Guinama (La Pobla de Vallbona, Spain) was used as reference vegetable oil to mix with the electrospun fiber mats to form gel-like dispersions. This vegetable oil is highly available, cost-effective, biodegradable, ecofriendly, and easy to extract from castor seeds [66]. Table 2 shows the fatty acid composition and main physical properties of the castor oil.

### 4.2. Preparation and Characterization of CTA Solutions

The study systematically investigated the effect of CTA concentration and binary solvent ratio (DCM/EtOH) on the morphology of electrospun fiber mats. CTA was dissolved in a binary solvent system comprising dichloromethane and ethanol (7/3 *v*/*v*) to obtain solutions with concentrations ranging from 1 to 7 wt.% after magnetic stirring at room temperature for 7 h. The impact of the DCM/EtOH solvent ratio on the most promising concentration was then studied in more detail. Therefore, DCM and EtOH were mixed in various compositional ratios (7/3, 8/2, 9/1, and 10/0 *v*/*v*) to investigate their effect on the morphology of the resultant CTA nanofibers.

The physico-chemical characteristics of CTA solutions were determined by measuring their electrical conductivity, surface tension, and shear viscosity. The electrical conductivity of the solution was measured using a Crison (GLP 31) conductivity meter (Crison, Barcelona, Spain). Prior to use, the conductivity cell was calibrated with standard KCl solutions of known conductivity (147 μS/cm, 1413 μS/cm, 12.88 μS/cm, and 111.8 μS/cm, respectively). The measurements were repeated at least three times to ensure accuracy. Surface tension measurements were conducted using a Wilhelmy platinum plate in a Sigma 703D tensiometer (Biolin Scientific, Beijing, China) with a measuring range of 1–1000 mN/m. The measurements were performed in duplicate at a temperature of 23 °C.

The shear rheological behavior of CTA solutions was characterized by applying a shear rate in the range of 1–500 s^−1^ at 23 °C using a controlled-strain rheometer (ARES Rheometric Scientific, Leatherhead, UK) equipped with coaxial cylinders (32 mm inner diameter, 1 mm gap, 33.5 mm length). CTA solutions with higher concentrations exhibited a non-Newtonian response, and the shear rate dependence of viscosity was fitted to the Carreau model (R^2^ > 0.995):

(9) $$\eta =\eta_{\infty }+\frac{\left(\eta_{0}-\eta_{\infty }\right)}{{\left[1+{\left(\frac{\dot{\gamma }}{{\dot{\gamma }}_{c}}\right)}^{2}\right]}^{p}}$$

where *η* is the non-Newtonian viscosity; $\eta_{0}\;$ and $\eta_{\infty }$ are the viscosities zero and infinite shear rates, respectively; $\dot{\gamma }$ is the shear rate; ${\dot{\gamma }}_{c}$ is the critical shear rate for the onset of the non-Newtonian region; and *p* is dimensionless constant which can be related to the exponent of the power law. 

### 4.3. Electrospinning Process and Characterization of CTA Electrospun Fibers

The electrospinning setup was designed using a high voltage power supply (Spellman, Hauppauge, NY, USA), a syringe with a capillary tip, a syringe pump (KD Scientific Inc.; Holliston, MA, USA), and a metallic grounded collector, all of which were placed in a sealed chamber. The CTA solution was loaded into a 10 mL syringe fitted with a stainless-steel needle (21G). The experiments were conducted with an applied field of 13–17 kV, a tip-to-collector distance of 15 cm, and a flow rate of 0.6–1 mL/h. All nanostructures were produced at room temperature (23 °C) and a relative humidity of 50%.

The morphology of the nanostructures was analyzed using a scanning electron microscope (SEM) model FlexSEM 1000 II (Hitachi, Tokyo, Japan) at 10 kV and various magnifications. Prior to imaging, samples were coated with a thin layer of gold using a sputter coater BTT150 (HHV, UK). The average diameter of the fibers was determined using image-analytical software (Image J 1.54g; NIMH, Bethesda, MD, USA). One hundred random observations were made with the same magnification for each sample.

### 4.4. Gel-like Dispersions Preparation

Gel-like dispersions were prepared by mixing selected electrospun CTA nanofibers with castor oil in an open batch reactor using an IKA RW-20 mixer (Staufen, Germany) with a low-shear anchor impeller geometry. The samples were processed for 1 h at room temperature (approximately 23 °C) and a rotational speed of 60 rpm. Following processing, all the samples were stored at room temperature for 2 days after processing for further rheological characterization. The prepared formulations were then stored for one month to check their physical stability. Visual inspection showed that these gel-like dispersions were physically stable with no signs of instability such as phase separation or loss of consistency. The gel-like dispersions were labelled based on the mass percentage of CTA nanofiber in the blend, the concentration of the spinning solution, and the binary solvent ratio. For example, 5CTA 70/30_4 wt.% represents samples thickened using 4 wt.% CTA nanofiber obtained from spinning solutions containing 5 wt.% CTA concentrations and a DM/EtOH 70/30 solvent ratio. 

### 4.5. Rheological Characterization of Gel-like Dispersions

Rheological tests were carried out in a controlled-stress rheometer Rheoscope (TermoHaake, Karlsruhe, Germany). Small amplitude oscillatory shear (SAOS) and steady-state flow measurements were performed. The SAOS tests were carried out in the linear viscoelastic region over a range of 0.02–100 rad/s under isothermal conditions, using a rough plate-and-plate geometry to avoid possible slip phenomena (20 mm, 1 mm gap, relative roughness 0.4). All rheological tests were performed at 23 °C.

### 4.6. Statistical Analysis

For each of the selected parameters, a statistical study was carried out. A one-way analysis of variance (ANOVA) was performed using two replicates of each measure independently. A number of statistical parameters were then calculated, including mean and standard deviation. In addition, a mean comparison test was performed to detect significant differences (*p* < 0.05).

## Figures and Tables

**Table 1:** Newtonian viscosity (*η*), non-Newtonian viscosities zero (*η_0_*), non-Newtonian viscosities infinite (*η*_∞_), critical shear rate (${\dot{\gamma }}_{c}$), and dimensionless constant (*p*) values of CTA spinning solutions in DCM/EtOH: 7/3.

Spinning Solutions	*η* (Pa∙s)	*η*_0_ (Pa∙s)	*η_∞_* (Pa∙s)	$\dot{\gamma }$_c_ (1/s)	*p* (-)
1 wt.% CTA	0.0072	-	-	-	-
1.5 wt.% CTA	0.0141	-	-	-	-
2 wt.% CTA	-	0.031	0.001	0.021	0.072
3 wt.% CTA	-	0.055	0.004	7.682	0.132
5 wt.% CTA	-	0.265	0.036	9.482	0.539
7 wt.% CTA	-	0.862	0.065	11.871	0.716

Note: Values differing in the superscripts are significantly different (*p* < 0.05).

**Table 2:** Physicochemical properties and fatty acid composition of vegetable oils (data from Martin-Alfonso et al. [67]).

Property	Castor Oil
Dynamic viscosity at 40 °C (mPa s)	230.0
Density at 15 °C (g/cm^3^)	0.9630
Myristic ^1^ 14:0 ^2^	-
Palmitic 16:0	1.70
Stearic 18:0	1.96
Oleic 18:1	5.34
Ricinoleic (18:1 -OH)	82.48
Linoleic 18:2	7.01
Linoleic 18:3	1.51
Saturated (SFAs)	3.66
Monounsaturated (MUFAs)	87.82
Polyunsaturated (PUFAs)	8.52
Unsaturated/saturated ratio	26.32

^1^ Fatty acid concentrations are in % of the total oil. ^2^ C:D, where C is the number of carbon atoms in the fatty acid chain, and D is the number of double bonds.

## Data Availability

The data presented in this study are openly available in the article.
